# Acupuncture combined with Chinese herbal medicine versus Chinese herbal medicine alone to improve clinical efficacy in treating endometriosis-associated pain: a systematic review and meta-analysis

**DOI:** 10.3389/fmed.2025.1649980

**Published:** 2025-10-16

**Authors:** Ziyi Xu, Nanzhu Wang, Junbo Liu, Changhui Li

**Affiliations:** ^1^College of Chinese Medicine, Changchun University of Chinese Medicine, Changchun, China; ^2^Department of Gynaecology, The Affiliated Hospital to Changchun University of Chinese Medicine, Changchun, Jilin, China

**Keywords:** endometriosis, acupuncture, Chinese herbal medicine, acupuncture combined with Chinese herbal medicine, randomized controlled trial

## Abstract

**Objective:**

This study aimed to assess the efficacy of acupuncture combined with Chinese herbal medicine (CHM) on endometriosis-associated pain.

**Methods:**

We searched eight electronic databases (PubMed, Web of Science, EMBASE, the Cochrane Library, CNKI, Wanfang, VIP, and SinoMed) to identify randomized controlled trials (RCTs) of acupuncture combined with CHM for endometriosis-associated pain. After literature screening and data extraction, statistical analysis was done with RevMan 5.4, and the risk of bias was assessed using the Cochrane Handbook’s Risk of Bias tool.

**Results:**

Our study included a total of 16 RCTs involving women with endometriosis-associated pain. Compared with CHM monotherapy, acupuncture combined with CHM significantly increased the clinical efficacy rate (OR = 3.75, 95% CI [2.58, 5.45], *p* < 0.00001) and reduced the visual analog scale (VAS) score (MD = −1.49, 95% CI [−2.43, −0.56], *p* < 0.0001).

**Conclusion:**

This systematic review indicates that acupuncture combined with CHM is a valuable non-hormonal option for endometriosis-related pain, outperforming CHM monotherapy in symptom relief and quality of life. It supports clinical integration, especially for patients unsuitable for hormonal therapies. However, conclusions are preliminary and require validation via large, rigorous RCTs, providing a reference for practice and future research.

**Systematic review registration:**

Identifier, CRD420250652517, https://www.crd.york.ac.uk/PROSPERO/.

## 1 Introduction

Endometriosis (EMS) is a common chronic condition in women, affecting 10% of women of reproductive age worldwide (1–3). Dysmenorrhea, pelvic pain, and infertility are common complications of this condition, which greatly reduce a woman’s quality of life (4). Moreover, recent research posits that women afflicted with endometriosis are predisposed to a heightened long-term risk of premature mortality, extending beyond their childbearing years (5). Endometriosis not only imposes a heavy health burden on the patient but also puts tremendous economic pressure on society. In the United States alone, it causes an economic loss of up to $22 billion annually (6).

Currently, pharmacological management of EMS primarily encompasses analgesics, non-steroidal anti-inflammatory drugs, and hormonal therapies (7–9). However, both clinical practice and translational research have revealed notable limitations in their clinical efficacy, and these agents are frequently associated with substantial adverse events. Among these interventions, progesterone therapy is disproportionately plagued by adverse reactions, leading to a treatment discontinuation rate as high as 25–50% (10, 11). Furthermore, long-term administration of hormonal therapy can not only induce mood disturbances but also elicit a spectrum of menopause-related symptoms (12). Against this backdrop, there is an urgent need to garner greater attention from the medical community and vigorously explore more effective alternative therapeutic strategies to enhance therapeutic outcomes, mitigate adverse events, and better address the unmet clinical needs of patients with EMS.

Chinese herbal medicine (CHM) plays an important role in improving pain (13), and acupuncture has advantages in the treatment of endometriosis-related pain (14, 15). The latest meta-analysis shows that acupuncture can effectively relieve dysmenorrhea and pelvic pain caused by the disease, improve the quality of life of patients, and reduce the recurrence rate (16). Another meta-analysis shows that Chinese patent medicine can be effective in treating EMS (15). As complementary medicine, acupuncture and CHM provide new ways to improve endometriosis pain (17, 18). However, a detailed, high-quality, and systematic methodological evaluation of acupuncture and CHM for endometriosis is lacking. Therefore, we conducted a new systematic evaluation for patients with endometriosis pain treated by CHM and acupuncture to answer the following clinical question:

1. Does the combination of CHM and acupuncture improve endometriosis pain compared to a single CHM treatment?

## 2 Methods

### 2.1 Strategy for literature search

#### 2.1.1 Data sources

This study searched eight databases from 2015 to 1 January 2025 [PubMed, Embase, Cochrane Central Register of Controlled Trials (CENTRAL), Web of Science (SCI), China Biomedical Database (CBM), China National Knowledge Infrastructure (CNKI), Wanfang Data Knowledge Service Platform, and VIP Journal Integration Platform (VIP)], which contain four English databases and four Chinese databases. The keywords used in the PubMed search included acupuncture, Chinese herbal medicine, Endometriosis, and randomized controlled trials (RCTs). See the Supplementary material for specific search strategies.

#### 2.1.2 Inclusion criteria

The inclusion criteria for this study are as follows:

1. Population: Studies that included adolescent or pre-menopausal women who were diagnosed with endometriosis according to symptoms, examination, biochemical results, imaging, laparoscopy, laparotomy, or visualization (with or without histological confirmation) were accepted.
2. Intervention: Acupuncture combined with CHM and the control group using CHM treatment.
3. Comparators: CHM treatment for endometriosis alone.
4. Outcomes: We considered the following outcome measures. The primary outcomes were dysmenorrhea and pelvic pain measured by the visual analog scale (VAS) and clinical effectiveness. Secondary outcomes included carbohydrate antigen (CA) 125 levels, Cox Menstrual Symptom Scale (CMSS), CHM syndrome scale, prostaglandin E2 (PGE2), and changes in posterior fornix tender nodules.
5. Study designs: RCTs.

#### 2.1.3 Exclusion criteria

The exclusion criteria for this study are as follows:

1. Participants: Studies were excluded when participants were diagnosed with primary dysmenorrhea, were postmenopausal, or when dysmenorrhea, pelvic pain, or dyspareunia were caused by inflammation, tumor, uterine myoma, or other causes. Studies that included participants who had undergone hysterectomy and/or unilateral or bilateral salpingo-oophorectomy for endometriosis, or bilateral salpingo-oophorectomy, were also excluded.
2. Interventions and comparators: Subjects receiving treatments other than acupuncture and CHM.
3. Outcomes: Studies that reported on outcomes other than those listed in Section 2.2.4 were ineligible.
4. Types of study: Non-randomized clinical trials, non-controlled studies, and studies involving animal or cell experiments were excluded.

### 2.2 Data abstraction and analysis

Two authors independently reviewed studies and extracted data according to the Preferred Reporting Items for Systematic Reviews and Meta-Analyses (PRISMA) guidelines. Any disagreements were resolved through discussion.

The methodological quality of the included RCTs was assessed using the Cochrane Risk of Bias Tool (19). (1) Random sequence generation (selection bias): Studies were rated as “low risk” if valid randomization methods (e.g., random number tables, computer-generated sequences) were explicitly described. Those merely stating “randomized” without specifying the method were rated as “unclear risk.” (2) Allocation concealment (selection bias): Trials were categorized as “low risk” if robust concealment strategies (e.g., central randomization, sealed opaque envelopes) were clearly reported. Insufficient details resulted in an “unclear risk” rating. (3) Blinding of participants and personnel (performance bias): For this domain, a “low risk” rating was assigned to studies where participants and research personnel were explicitly confirmed to be unaware of the intervention type received by participants. A “high risk” rating was given if there was clear evidence confirming that participants or research personnel had awareness of the intervention type. An “unclear risk” rating was determined when studies provided inadequate reporting of details regarding the blinding status of participants and research personnel, making it impossible to verify whether blinding was implemented. (4) Blinding of outcome assessment (detection bias): Emphasis was placed on whether outcome assessors were blinded, particularly for subjective endpoints such as pain scores (VAS). Studies failing to report assessor blinding were rated as “unclear risk.” (5) Incomplete outcome data (attrition bias): “Low risk” was assigned if missing data were balanced between groups and appropriately handled (e.g., intention-to-treat analysis). Studies with unaddressed attrition or inadequate explanations were rated as “high risk.” (6) Selective reporting (reporting bias): Evaluated by comparing available study protocols with published results. Consistency between pre-specified and reported outcomes resulted in “low risk”; otherwise, “unclear risk” was assigned. (7) Other biases: This included assessment of baseline comparability between groups, funding sources, and other methodological issues potentially threatening validity.

Review Manager (RevMan, version 5.4.1, Cochrane Collaboration) was used for the planned meta-analysis using a random-effects model. We planned to analyze continuous data using mean difference (MD) or standardized mean difference (SMD) with 95% confidence intervals (CI) with an inverse variance. For dichotomous data, we planned to calculate the risk ratio (RR) with 95% CI using Mantel–Haenszel variance. However, clinical heterogeneity was detected in the intervention types, participants, and treatment duration that prevented meta-analysis. The results for each planned comparison and outcome were obtained using available case analysis. A *p*-value < 0.05 was considered statistically significant.

## 3 Results

### 3.1 Study search and description

**Figure 1:**
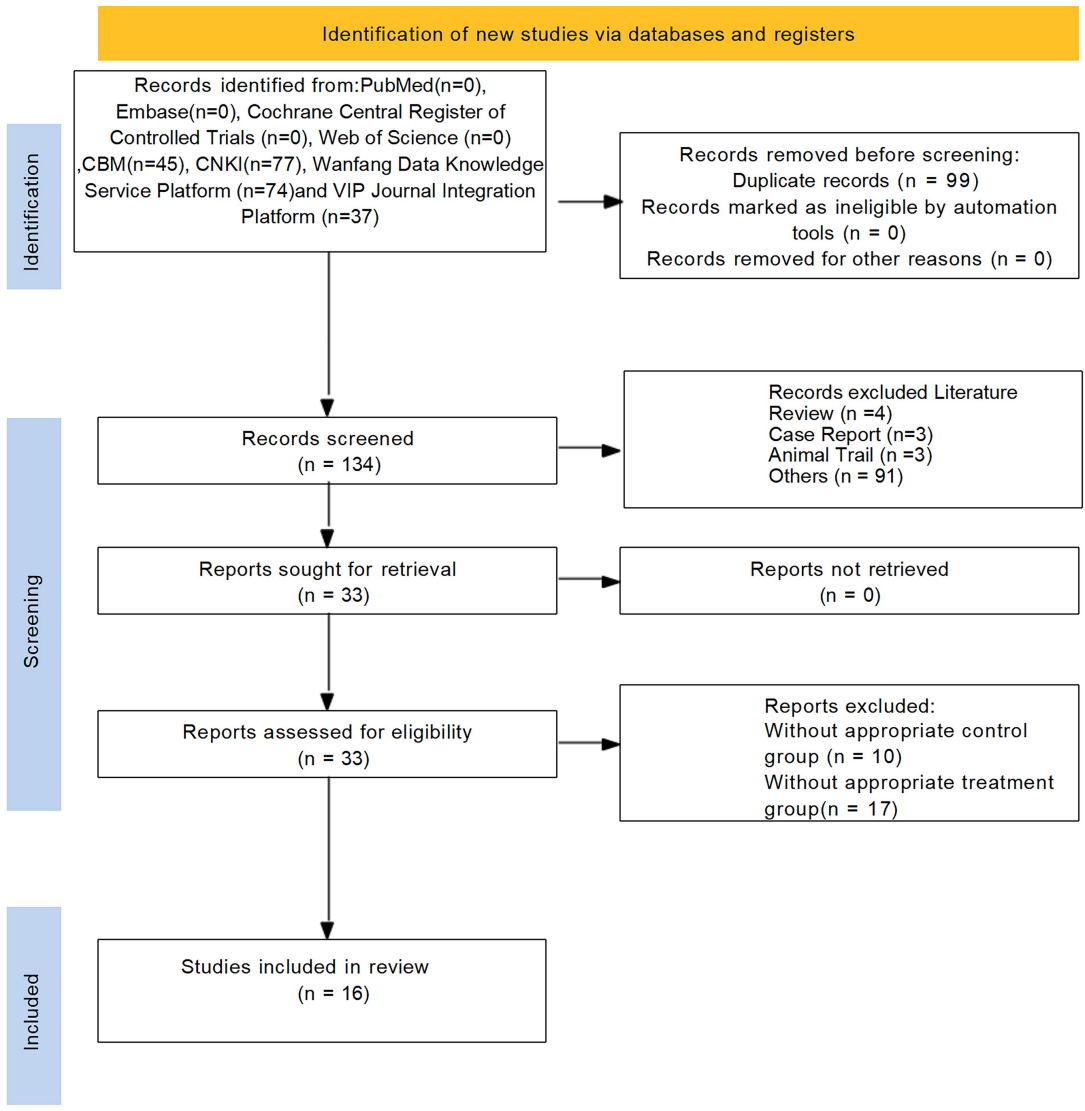
Flowchart of the study selection process.

Initially, a total of 233 studies were included according to the search strategy. After removing 99 duplicate entries, 101 studies were removed based on title and abstract. After a comprehensive screening of the full text, 16 studies were finally included in the full text. The flowchart of the literature screening process is shown in Figure 1.

### 3.2 Characteristics of the included studies

A total of 16 RCTs were included in this systematic review, all of which were conducted in China (20–35). The studies predominantly focused on endometriosis-related dysmenorrhea (15 trials), with only one investigation addressing chronic pelvic pain associated with endometriosis (26). The sample sizes ranged from 54 to 146 participants, with patient ages typically falling between 21 and 55 years across studies. The reported disease duration varied from approximately 1–10 years. The control groups received CHM alone in most studies, with one trial using a combination of Comfort injection and CHM (23). Follow-up periods were explicitly reported in 15 studies, ranging from 1 to 6 months post-treatment. Basic characteristics of the included studies are shown in Table 1.

**Table 1:** Characteristics of the 16 trials identified in the literature search.

	Study type	Sample size	Age (T/C)	Clinical diagnosis (pain type)	Course of disease	Intervention	Course of treatment	Follow-up visit
Huang LR 2020 (25)	Monocentric	27/27	29.67 ± 3.58/28.93 ± 3.33	Menstrual pain	4.59 ± 1.89/4.13 ± 1.71	Acupuncture + Chinese herbal medicine	Chinese herbal medicine	3 months
Ni JF 2023 (27)	Monocentric	73/73	33.15 ± 3.05/32.31 ± 3.63	Menstrual pain	**/**	Acupuncture + Chinese herbal medicine	Chinese herbal medicine	**/**
Zhang XH 2017 (33)	Monocentric	45/45	22.80 ± 7.20/21.70 ± 6.30	Menstrual pain	5.80 ± 3.40/5.90 ± 4.10	Acupuncture + Chinese herbal medicine	Chinese herbal medicine	3 months
Niu XX 2012 (30)	Monocentric	33/29	23.65	Menstrual pain	1–7	Acupuncture + Chinese herbal medicine	Chinese herbal medicine	3 months
Luo YF 2022 (24)	Monocentric	45/45	30.52 ± 1.08/30.32 ± 1.13	Menstrual pain	2.32 ± 0.46/2.21 ± 0.35	Acupuncture + Chinese herbal medicine	Chinese herbal medicine	3 months
Tian LY 2016 (31)	Monocentric	35/35	34.86 ± 6.13/33.06 ± 7.12	Menstrual pain	3.82 ± 2.46/4.06 ± 2.46	Acupuncture + Chinese herbal medicine	Chinese herbal medicine	4 months
Xiang DF 2011 (34)	Monocentric	38/30	34.51 ± 5.71/33.34 ± 5.01	Menstrual pain	2.87 ± 1.21/2.80 ± 1.05	Acupuncture + Chinese herbal medicine	Chinese herbal medicine	3 months
Zhao JQ 2023 (28)	Monocentric	40/40	36.98 ± 5.32/ 36.58 ± 3.84	Menstrual pain	3.93 ± 2.2/4.30 ± 2.09	Acupuncture + Chinese herbal medicine	Chinese herbal medicine	3 months
Gao Q 2021 (21)	Monocentric	36/36	37.23 ± 4.83/36.89 ± 5.27	Menstrual pain	3.64 ± 2.21/3.73 ± 2.46	Acupuncture + Chinese herbal medicine	Chinese herbal medicine	3 months
Shen GL 2023 (22)	Monocentric	40/40	28.64 ± 2.52/28.37 ± 2.34	Menstrual pain	3.34 ± 0.017/3.47 ± 0.25	Acupuncture + Chinese herbal medicine	Chinese herbal medicine	6 months
Wang YJ 2018 (20)	Monocentric	40/40	37.8 ± 3.5/ 35.2 ± 3.6	Menstrual pain	2.7 ± 0.4/2.6 ± 0.5	Acupuncture + Chinese herbal medicine	Chinese herbal medicine	3 months
Liu QL 2018 (29)	Monocentric	29/29	35.2 ± 5.5/34.7 ± 5.3	Menstrual pain	6.7 ± 2.6/6.4 ± 2.3	Acupuncture + Chinese herbal medicine	Chinese herbal medicine	3 months
Zou SN 2023 (32)	Monocentric	49/47	32.24 ± 4.51/31.83 ± 4.68	Menstrual pain	**/**	Acupuncture + Chinese herbal medicine	Chinese herbal medicine	3 months
Ma FY 2021 (26)	Monocentric	30/30	34.5 ± 6.3	Chronic pelvic pain	5.1 ± 2.3	Acupuncture + Chinese herbal medicine	Chinese herbal medicine	2 months
Sun KF 2023 (23)	Monocentric	36/34	27.21 ± 4.89/26.2 ± 5.31	Menstrual pain	5.3 ± 2.19/3.45 ± 1.56	Acupuncture + Chinese herbal medicine	Comfort injection+ Chinese herbal medicine	3 months
Yang DX 2015 (35)	Monocentric	21/21/21	21–55	Menstrual pain	2–10	Acupuncture + Chinese herbal medicine	Chinese herbal medicine	3 months

### 3.3 Quality assessment of included studies

**Figure 2:**
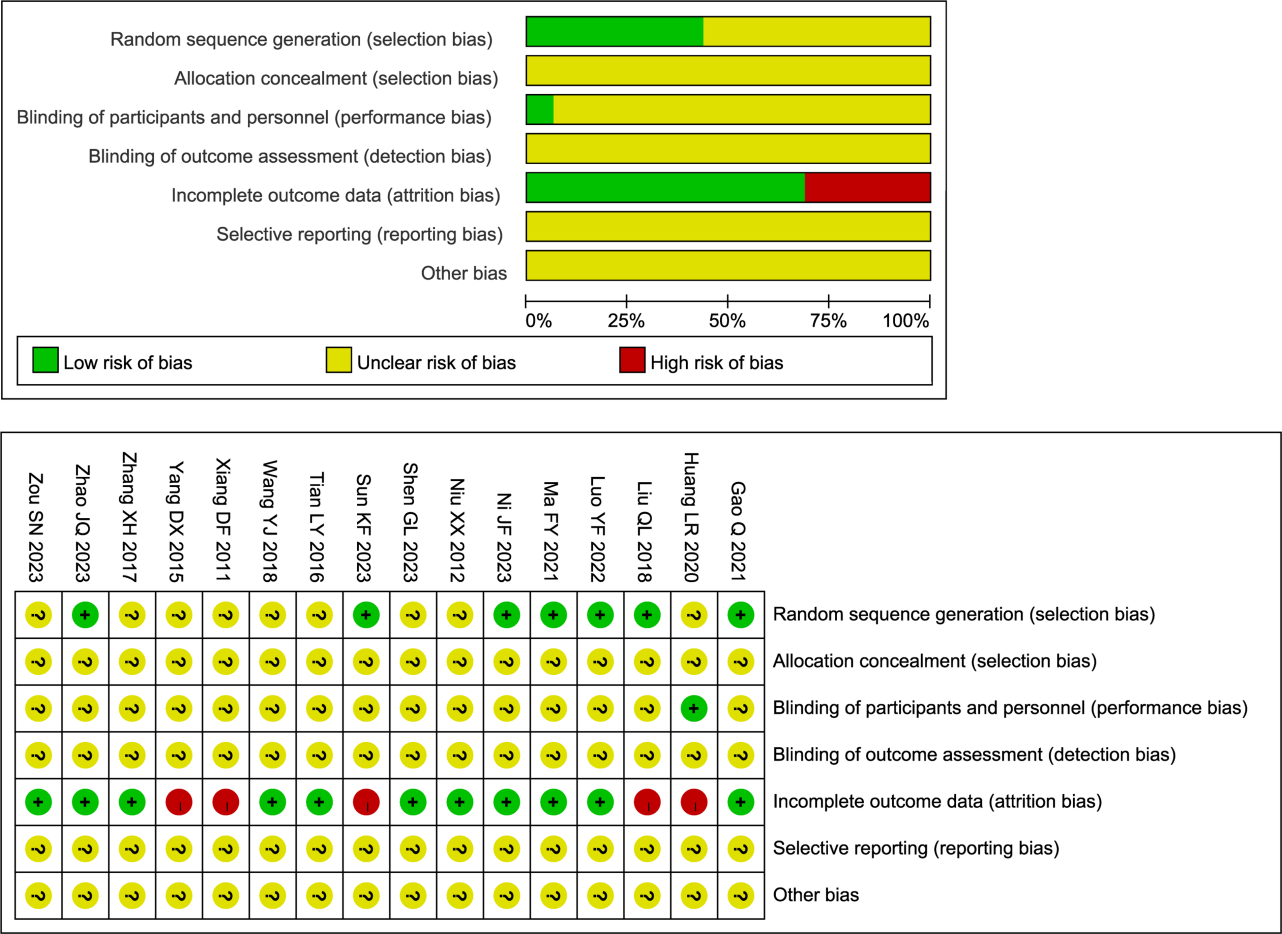
Risk of bias assessment graph of the included studies.

The methodological quality of the included RCTs was evaluated using the Cochrane Risk of Bias tool. The overall results are presented in Figure 2. Regarding random sequence generation, seven studies clearly described the method used. Among these, six studies utilized a random number table (21, 23, 26–29) and one employed a lottery method (24); all were rated as low risk of bias for this domain. The remaining nine studies only stated that participants were “randomly assigned” without specifying the method, resulting in an unclear risk of bias. Allocation concealment was inadequately reported across all studies. None of the 16 trials described methods to conceal the allocation sequence from investigators and participants prior to assignment. Consequently, the risk of bias for this domain was judged as unclear for all included studies. In addition, five studies reported participant withdrawals and were rated as high risk of bias due to attrition (23, 25, 29, 34, 35). Moreover, blinding of outcome assessors was rarely described across the studies. Only one trial explicitly reported the use of a single-blind design (25); for all other trials, the blinding status could not be determined due to insufficient reporting. Other potential sources of bias, including selective reporting, remained unclear due to insufficient information. Detailed judgments for each study are available in Figure 2.

### 3.4 Clinical outcomes of meta-analysis

#### 3.4.1 Main outcome indicators

##### 3.4.1.1 Variation in pain level

**Figure 3:**
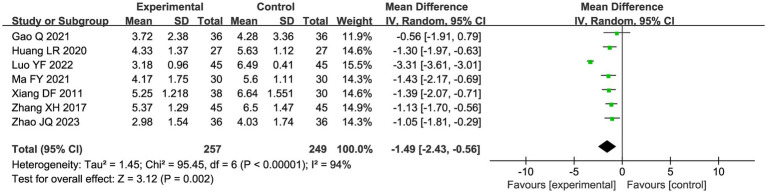
Forest plot of VAS scores meta-analysis.

**Figure 4:**
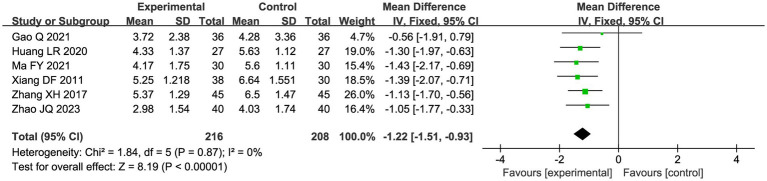
Sensitivity analysis graph of VAS scores after excluding Luo (24).

**Figure 5:**
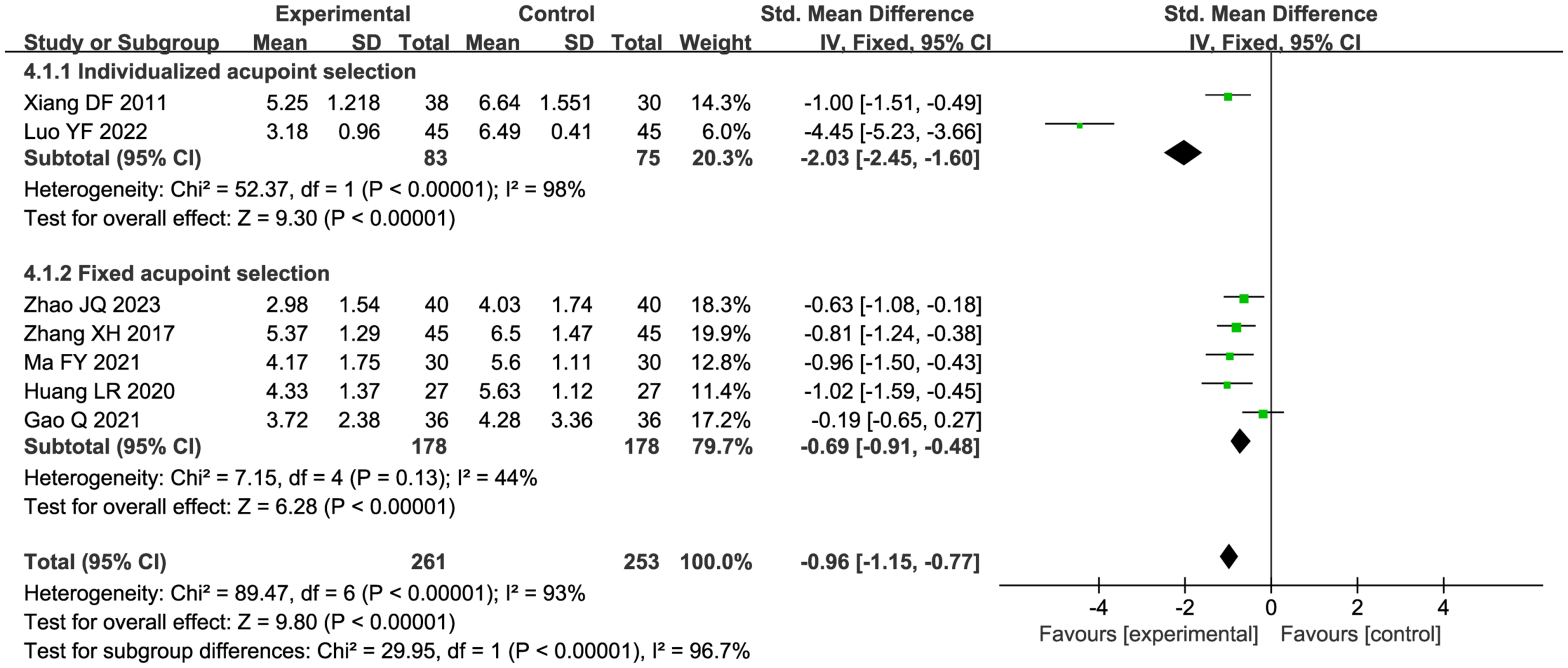
Subgroup analysis forest plot of VAS scores by acupoint selection.

A total of seven studies compared the difference in pain levels between the CHM group and the acupuncture CHM combination group. Figure 3 showed that the VAS score was significantly lower in the combined treatment group (MD = −1.49, 95%CI: [−2.43, −0.56], *p* = 0.002), but there was high heterogeneity (I^2^ = 94%, *p* < 0.00001), so we chose a random effects model for meta-analysis. By sensitivity analysis and by excluding the Luo YF (2022) study (24), the heterogeneity disappeared completely (I^2^ = 0%) in Figure 4. The direction of the effect sizes remained consistent (MD = −1.49 versus −1.22), indicating robust results. To further explore the reasons for the high heterogeneity, we performed subgroup analysis according to different acupuncture acupoint selection methods in Figure 5. The combined results of five fixed selection studies showed significant efficacy (SMD = −0.69, 95%CI [−0.91, −0.48]) with low heterogeneity (I^2^ = 44%). Two studies of personalized acupoint selection were not combined due to the principal differences in acupoint selection and the high heterogeneity (I^2^ = 98%) (24, 34). Among them, the effect size of Luo YF (2022) was the largest (SMD= −4.45, 95% CI: −5.23 to −3.66) (24), suggesting that accurate traditional Chinese medicine (TCM) pattern differentiation may be a critical factor in enhancing the efficacy of acupuncture.

##### 3.4.1.2 Response rate

**Figure 6:**
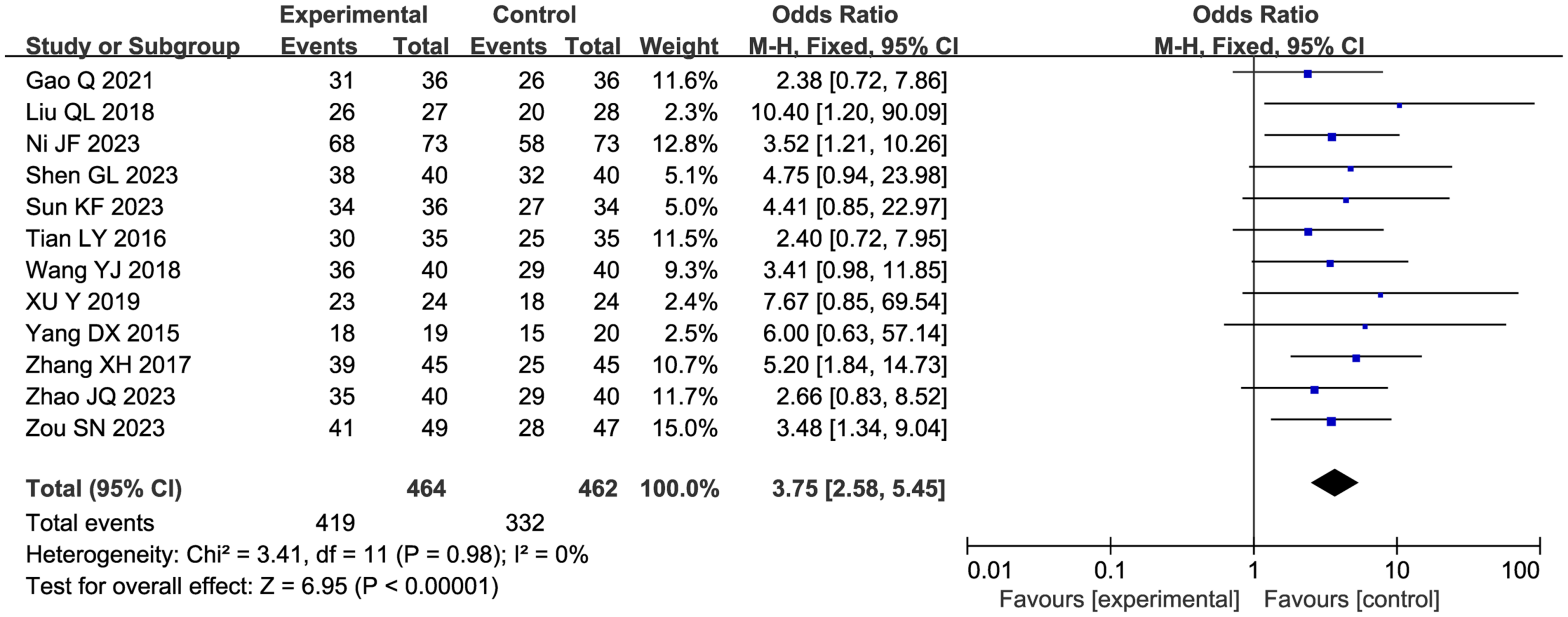
Forest plot of clinical response rate meta-analysis.

A total of 12 studies reported a total clinical response rate, and the combined results showed a slight heterogeneity (I^2^ = 0%), so a fixed-effect model was used in Figure 6. Meta-analysis showed that acupuncture combined with CHM had a positive clinical response effect in patients with endometriosis-related pain compared with the CHM group (OR 3.75, 95% CI: [2.58, 5.45], *p* < 0.00001).

#### 3.4.2 Secondary outcomes

##### 3.4.2.1 Variation in serum CA-125 levels

**Figure 7:**
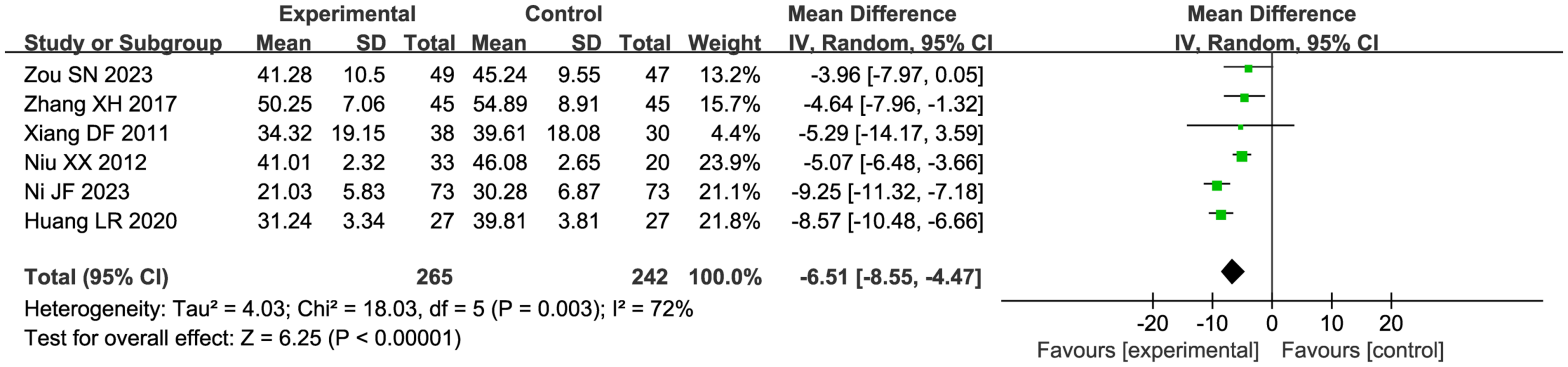
Forest plot of serum CA-125 levels meta-analysis.

Six studies compared the changes in serum CA-125 levels in patients in the acupuncture combination and CHM group in Figure 7. The results of the meta-analysis showed significant heterogeneity (I^2^ = 72%), so we used a random effect model for the analysis. Compared with CHM alone, the combination of acupuncture and CHM had a positive effect on reducing serum CA-125 (MD = −6.51, 95% CI −8.55, −4.47, *p <* 0.00001).

##### 3.4.2.2 Scores under the new drug guidelines and CMSS

**Figure 8:**
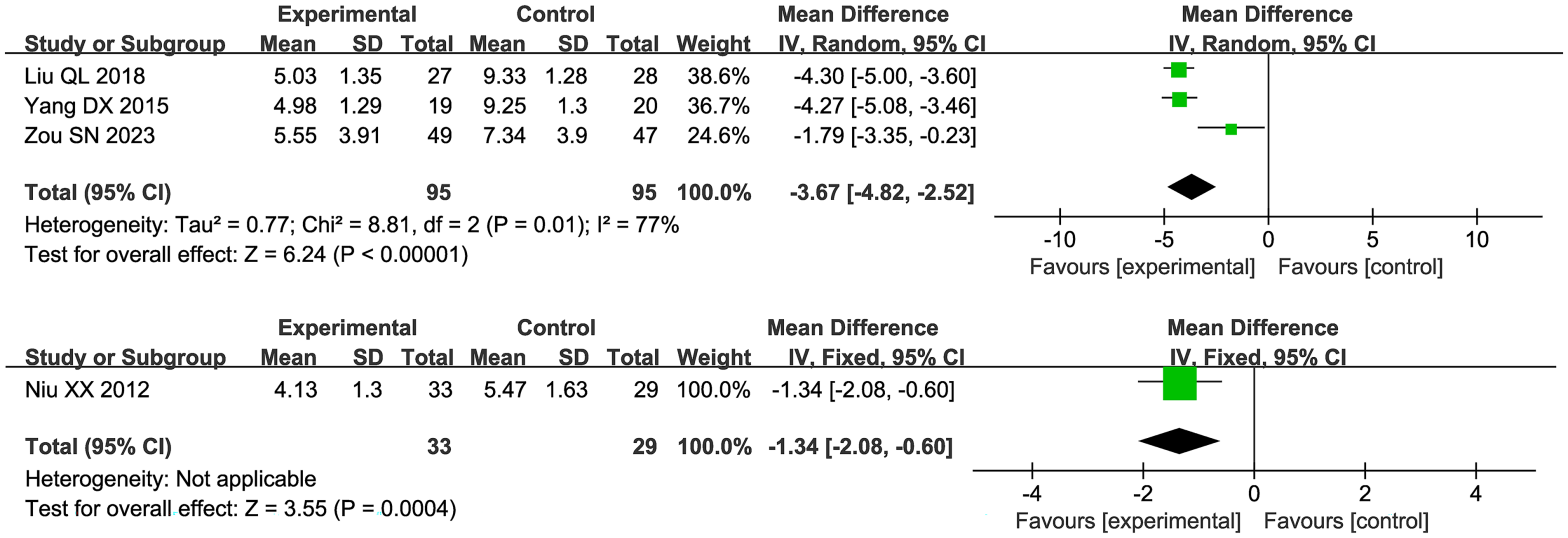
Forest plot of new drug guidelines scores & CMSS meta-analysis.

Four studies reported scores under the new drug guidelines, and due to high heterogeneity (I^2^ = 77%), a random effects model was used in Figure 8. The results showed that the combination of acupuncture and Chinese herbs had good pain relief compared to the control group (MD −3.67 95% [−4.82, −2.52]). The study by Niu et al. used CMSS, and compared with the control group, the trial group had CMSS (MD −1.34 95% [−2.08, −0.60]).

##### 3.4.2.3 CHM syndrome scale

**Figure 9:**
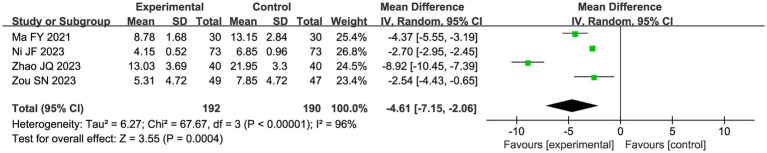
Forest plot of CHM syndrome scale scores meta-analysis.

Four studies evaluated the CHM syndrome scale using a random effects model due to the apparent heterogeneity (I^2^ = 96%). Meta-analysis showed that the CHM combination and acupuncture significantly improved the CHM syndrome scale compared with the control group (MD 4.61, 95%CI: −7.15, 2.06, *p* = 0.0003; Figure 9).

##### 3.4.2.4 PGE2

**Figure 10:**
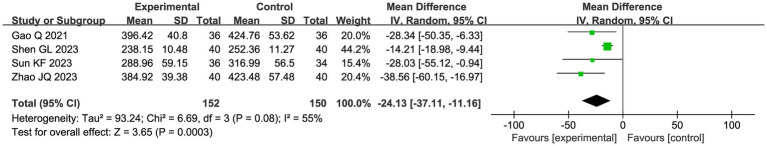
Forest plot of PGE2 levels meta-analysis.

Four studies evaluated the assessment of PGE 2, applying a random effects model due to heterogeneity (I^2^ = 55%). The meta-analysis of the pooled data showed that combining CHM with acupuncture as an adjuvant intervention significantly reduced PGE 2 compared with the control group (MD −24.13, 95% CI: −37.22, −11.16, *p* = 0.0003; Figure 10).

##### 3.4.2.5 Posterior fornix tender nodules changes

**Figure 11:**
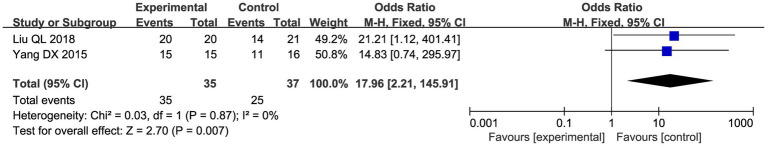
Forest plot of posterior fornix tender nodules improvement meta-analysis.

Two studies evaluated the improvement in vault tender nodule pain after treatment. The results of the meta-analysis showed that CHM and acupuncture significantly improved posterior vault tender nodule pain compared with the control group (OR = 17.96, 95% CI: 2.21–145.91, *p* = 0.007; Figure 11).

### 3.5 Publication bias

**Figure 12:**
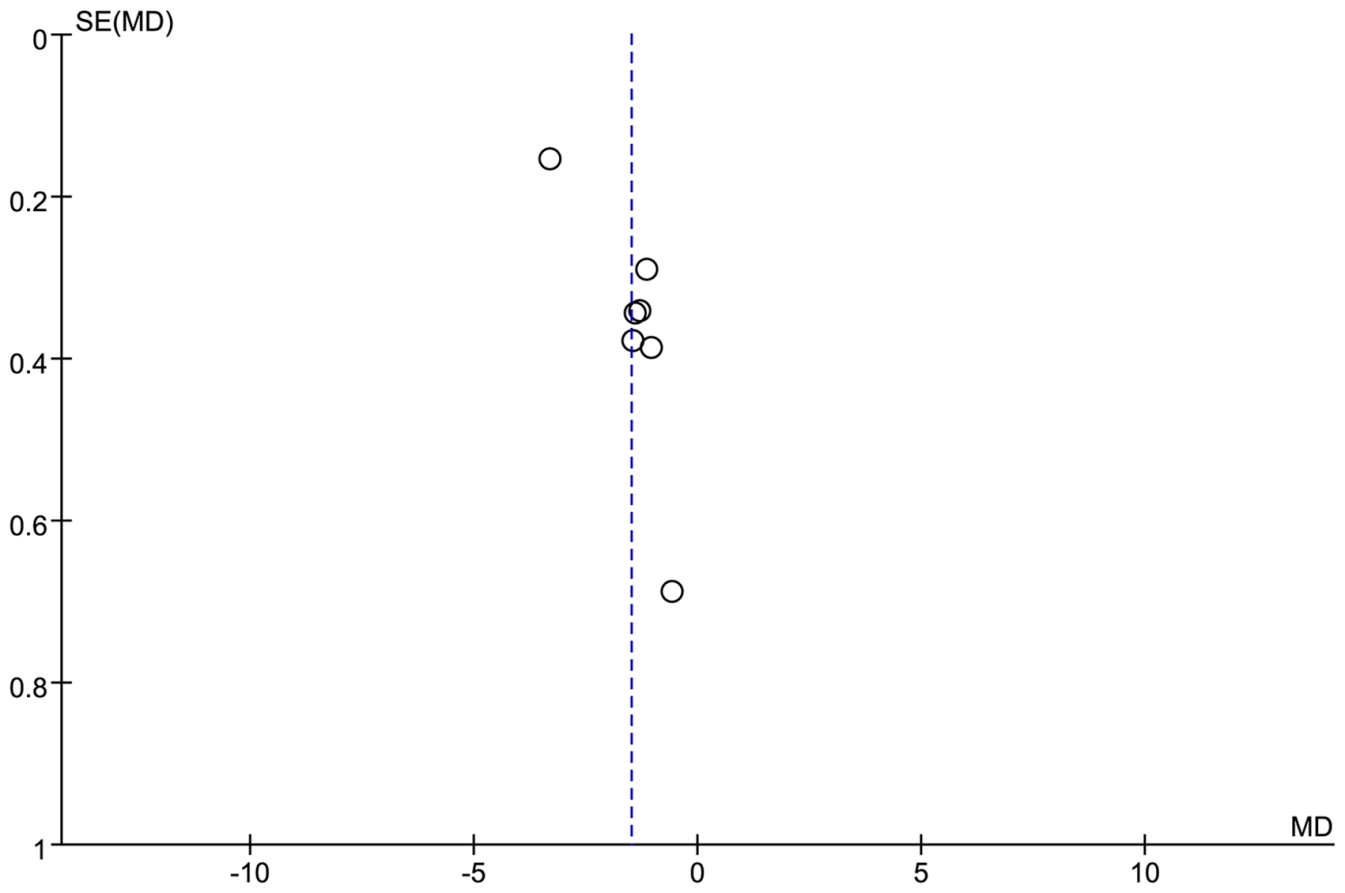
Funnel plot for publication bias assessment of VAS scores meta-analysis.

To assess the potential publication bias of the VAS score (Figure 12), this study was visually analyzed by funnel plots. The results showed that the funnel plot showed good symmetry of the effect size distribution. The results showed that the effect size point estimates for the included studies were approximately symmetrically distributed around the pooled effect line. However, it should be emphasized that the symmetry of the funnel plot can only provide preliminary visual interpretation evidence, and it is suggested to expand the sample size in future studies.

## 4 Discussion

Endometriosis causes significant pain and fertility burden to women and greatly reduces their quality of life (36). Currently, traditional laparoscopic surgery and hormonal treatments are widely used, but they suffer from high recurrence rates and obvious side effects (37). Acupuncture and CHM, as complementary medicine, have shown potential advantages in relieving pain caused by endometriosis (17, 18). However, comprehensive evidence on the combined use of acupuncture and herbal medicine is still relatively limited. To the best of our knowledge, this is the first systematic evaluation and meta-analysis to assess the effectiveness of the combination of acupuncture and Chinese herbs for the treatment of endometriosis.

In this study, we included a total of 16 RCTs to systematically compare the difference in efficacy between the acupuncture–herb combination therapy and single herbal intervention. The results of the meta-analysis showed that acupuncture combined with CHM significantly increased the clinical efficacy rate(OR 3.75, 95% CI: [2.58, 5.45], *p* < 0.00001) and decreased the VAS score (MD= −1.49, 95% CI: [−2.12, −0.86], *p* < 0.0001) compared with single herbal treatment. In addition to this, significant improvements were observed in posterior fornix tenderness nodules, inflammatory factor levels, CHM evidence scores, and quality of life scores. The combined use of Chinese herbs and acupuncture combines internal and external therapies, and compared with single herbal treatment, acupuncture acts directly on the pain site to rapidly relieve pain symptoms by regulating local qi and blood flow and inhibiting inflammatory responses (38). Clinical studies have shown that the efficacy of acupuncture is weakened after withdrawal (38), while CHM can prolong the treatment effect and reduce the recurrence of symptoms by continuously regulating the internal environment (18).

The results of subgroup analysis based on different acupuncture methods show that the acupuncture method using individualized syndrome differentiation has significant advantages in efficacy, which provides a scientific basis for optimizing the acupuncture treatment scheme. The core of TCM is “treatment based on syndrome differentiation” (39). This finding implies that the efficacy of acupuncture, and potentially herbal medicine, is not merely a function of the intervention itself but is significantly modulated by the accuracy of the CHM pattern diagnosis. Future clinical trials of CHM therapies should prioritize the incorporation and detailed reporting of pattern differentiation protocols to ensure treatment fidelity and maximize therapeutic outcomes. Whether it is internal treatment of Chinese medicine or external treatment of acupuncture, precise treatment strategies should be formulated according to individual differences of patients (such as syndrome type, etiology, and pathogenesis) (40).

The pathogenesis of endometriosis is complex, including chronic inflammatory response, oxidative stress, and immune regulation imbalance, and the inflammatory response is the main factor of endometriosis-related pain (41, 42). The results of our meta-analysis indicate that acupuncture combined with CHM treatment has significant efficacy in improving tenderness nodules, reducing the level of inflammatory factors, and relieving pain, and its mechanism of action may include the following aspects. CHM regulates inflammation-related signaling pathways through multiple components and targets to inhibit the release of pro-inflammatory factors (such as TNF-*α*, IL-6, and IL-1β), thus reducing local and systemic inflammatory responses (43, 44). Acupuncture has a good analgesic effect and exerts synergistic anti-inflammatory effects by stimulating specific acupoints and regulating the neuro–endocrine–immune network to further inhibit the production of inflammatory mediators (45–47). CHM regulates the qi, blood, and Yin and Yang of the body, and improves the internal environment (48), while acupuncture directly acts on the lesions through local stimulation (49, 50). The combination of the two can realize multi-way and multi-target synergistic treatment. This mode of “global regulation + local treatment” can not only significantly relieve the pain but also delay the disease progression and improve the quality of life of patients by regulating the immune function and neuroendocrine system (51, 52).

Previous meta-analyses mostly focused on the efficacy evaluation of a single intervention measure (e.g., CHM or acupuncture) and failed to fully assess the advantages of combination therapy. Although some studies have explored the effects of combining CHM and acupuncture, their conclusions are controversial. A meta-analysis by Xiao et al. showed a statistically insignificant effect of acupuncture combined with Chinese herbs in improving endometriosis-related pain (53); a study by Chen et al. reached a similar conclusion (16). However, these studies have some limitations, such as early publication and a limited number of RCTs included, which may lead to insufficient statistical efficacy and biased results. The results of this study are consistent with the network meta-analysis by Su et al., which confirmed that acupuncture combined with Chinese herbs can significantly improve endometriosis-related pain (17).

Our study provides a systematic assessment of the effectiveness of CHM combined with acupuncture for the treatment of dysmenorrhea in endometriosis, but there are still limitations. First, the small sample size of the included RCT studies may undermine the reliability of the results. Second, most of the included studies did not mention the implementation of allocation concealment and blinding, which may overestimate the treatment benefit. In addition to this, the CHM symptom score and quality of life scale used inconsistent criteria for evaluating efficacy across studies. Finally, all the included studies were from the Chinese-language literature, which may be subject to geographic bias. In addition, although the funnel plot did not suggest significant publication bias, the possibility that negative results were not published can still not be completely excluded. Furthermore, a significant limitation is that the majority of included studies either lacked adequate reporting of TCM pattern diagnosis methodology or did not apply it at all. The absence of standardized implementation and reporting of pattern differentiation introduces substantial heterogeneity, which may compromise the interpretation and generalizability of our findings. These limitations somewhat undermine the reliability of the evidence presented in the current study, and thus, further validation is needed to draw conclusive conclusions. Based on these limitations, we need a more well-designed, high-quality, large-sample-size RCTs in the future in light of these limitations to consolidate confidence in the benefits of combination therapy for dysmenorrhea. It is expected that future work will incorporate more multicenter, large-sample, and well-designed RCTs for different types of endometriosis-related pain and various acupuncture techniques.

## 5 Conclusion

This systematic review and meta-analysis suggest that combining acupuncture with CHM may offer potential benefits in managing endometriosis-related pain compared to herbal medicine alone. The evidence indicates that the integrative approach could enhance overall efficacy, alleviate symptoms, and improve quality of life, supporting its consideration as a non-hormonal treatment alternative, particularly for patients with contraindications or inadequate response to conventional therapies. These findings highlight the need for further research into the integration of acupuncture into existing therapeutic frameworks and may inform clinical decision-making for patients seeking alternatives to hormonal interventions. However, these conclusions are constrained by the methodological limitations and the limited number of included trials. Therefore, high-quality, large-scale RCTs are imperative to confirm these preliminary findings and establish robust evidence for clinical practice.

## Data Availability

The original contributions presented in the study are included in the article/Supplementary material, further inquiries can be directed to the corresponding author.
