# Supplementary material for: Acupuncture combined with Chinese herbal medicine versus Chinese herbal medicine alone to improve clinical efficacy in treating endometriosis-associated pain: a systematic review and meta-analysis
Source: Front Med (Lausanne). 2025 Oct 16;12:1649980. doi: 10.3389/fmed.2025.1649980 (PMC12571871; doi:10.3389/fmed.2025.1649980)
Supplement: Supplementary file 1 [file Data_Sheet_1.pdf]

Strategy for literature search.

PubMed

1#Acupuncture*[Title/Abstract] OR Needling[Title/Abstract] OR Electroacupuncture*[Title/Abstract] OR Electro-acupuncture[Title/Abstract] OR Acupoint Therapy[Title/Abstract] OR Acupuncture Treatment[Title/Abstract] OR AcupunctureTreatments[Title/Abstract] OR Needle Therapy[Title/Abstract] OR silver needle[Title/Abstract] OR de qi[Title/Abstract] OR needle pricking[Title/Abstract] OR needling[Title/Abstract]

2#Chinese herbal medicine”[Ti/Ab] OR “CHM”[Ti/Ab] OR “traditional Chinese medicine”[Ti/Ab] OR “TCM”[Ti/Ab] OR “herbals”[Ti/Ab] OR “herbal medicine” [Ti/Ab] OR “Chinese Medicine” [Ti/Ab]

3# 1# AND 2#

4#Endometriosis[MeSH Terms] OR Endometriosis[Title/Abstract] OR Endometriosis*[Title/Abstract] OR Endometrioses[Title/Abstract] OR Endometrioma[Title/Abstract] OR Endometriomas[Title/Abstract] OR EMs[Title/Abstract]

5# Randomized Controlled Trial[Publication Type] OR Randomized Controlled Trials as Topic[MeSH Terms] OR Randomized Controlled Trial[All Fields] OR RCT[All Fields] OR
Trial*[All Fields] OR random*[Title/Abstract]

6# #1 AND #4 AND #5

EMBASE

#1 'Acupuncture'/exp

#2 'Acupuncture Points'/exp

#3 'Acupuncture Analgesia'/exp

#4 'Acupuncture Therapy'/exp

#5'acupuncture*':ti,ab,kw OR 'electroacupuncture*':ti,ab,kw OR 'electro-acupuncture':ti,ab,kw OR 'Acupuncture Treatment':ti,ab,kw OR 'Acupuncture Treatments':ti,ab,kw OR 'needle therapy':ti,ab,kw OR 'silver needle':ti,ab,kw OR 'de qi':ti,ab,kw

#6 'Acupuncture Therapy'/exp

#7 #1 OR #2 OR #3 OR #4 OR #5 OR #6

#8'Chinese herbal medicine'/exp

#9' Chinese herbal medicine':ti,ab,kw OR' CHM* ':ti,ab,kw OR 'traditional Chinese medicine':ti,ab,kw OR'TCM':ti,ab,kw OR' herbals* ':ti,ab,kw OR 'herbal medicine':ti,ab,kw OR 'Chinese Medicine':ti,ab,kw

#10 #8 OR #9

#11 #10 AND #7

#12 ' Endometriosis '/exp

#13 ' Endometriosis ':ti,ab,kw OR ' Endometriosis* ':ti,ab,kw OR ' Endometrioses ':ti,ab,kw OR '
Endometrioma ':ti,ab,kw OR ' Endometriomas ':ti,ab,kw OR ' EMs ':ti,ab,kw

#14 #12 OR #13

#15 #14 AND #11

#16 'randomized controlled trial'/exp OR 'randomized controlled trial (topic)'/exp OR
random*:ti,ab,kw OR 'RCT':ti,ab,kw OR Trial*:ti,ab,kw
#17 #15 AND #16

Cochrane Library

#1 MeSH descriptor: [Acupuncture] explode all trees
#2 MeSH descriptor: [Acupuncture Points] explode all trees
#3 MeSH descriptor: [Acupuncture Analgesia] explode all trees
#4 MeSH descriptor: [Acupuncture Therapy] explode all trees
#5 (acupuncture*):ti,ab,kw OR (electroacupuncture*):ti,ab,kw OR (electro-acupuncture):ti,ab,kw OR (acupuncture*):ti,ab,kw OR (electroacupuncture*):ti,ab,kw OR (electro-acupuncture):ti,ab,kw OR (Acupuncture Treatment):ti,ab,kw OR
(Acupuncture Treatments):ti,ab,kw OR (needle therapy):ti,ab,kw OR (silver needle):ti,ab,kw OR (de qi):ti,ab,kw
#6 #1 OR #2 OR #3 OR #4 OR #5
#7 MeSH descriptor: [Chinese herbal medicine] explode all trees
#8' (Chinese herbal medicine)':ti,ab,kw OR' (CHM*) ':ti,ab,kw OR (traditional Chinese medicine)':ti,ab,kw OR (TCM):ti,ab,kw OR' (herbals*) ':ti,ab,kw OR (herbal medicine):ti,ab,kw OR (Chinese Medicin):ti,ab,kw
#9 #8 OR #7
#10 #9 AND #6
#11 MeSH descriptor: [Endometriosis] explode all trees
#12 (Endometriosis):ti,ab,kw OR (Endometriosis*):ti,ab,kw OR (Endometrioses):ti,ab,kw OR
(Endometrioma):ti,ab,kw OR (Endometriomas):ti,ab,kw OR (EMs):ti,ab,kw
#13 #11 OR #12
#14 #10 AND #13
#15 MeSH descriptor: [Randomized Controlled Trial] explode all trees
#16 MeSH descriptor: [Randomized Controlled Trials as Topic] explode all trees
#17 (random*):ti,ab,kw OR (RCT):ti,ab,kw OR (Trial*):ti,ab,kw
#18 #15 OR #16 OR #17
#17 #14 AND #18

Web of Science

#1 TS=(Chinese herbal medicine)) OR TS=(CHM) OR TS=(traditional Chinese medicine) OR TS=(TCM) OR TS=(herbals)) OR TS=(herbal medicine) OR TS=(Chinese Medicine)
#2 TS=(acupuncture*) OR TS=(electroacupuncture*) OR
TS=(electro-acupuncture) OR TS=(acupoint therapy) OR TS=(Acupuncture Treatment) OR TS=(Acupuncture Treatments) OR TS=(needle therapy) OR TS=(silver needle) OR TS=(de qi) OR TS=(meridian) OR TS=(needle pricking) OR TS=(needling)
#3 #1AND #2

#4 TS=(Endometriosis) OR TS=(Endometriosis*) OR TS=(Endometrioses) OR TS=(Endometrioma) OR TS=(Endometriomas) OR TS=(EMs)
#5 #3 AND #4
#6 TS=(Randomized Controlled Trial) OR TS=(Randomized Controlled Trials as Topic) OR TS=(Randomized Controlled Trials as Topic) OR TS=(random*) OR TS=(RCT) OR TS=(Trial*)
#7 #5 AND #6

China National Knowledge Infrastructure (CNKI)

SU='zhenjiu'+'zhenci'+'dianzhen'+'tang'+'zhongyao'+'zhenyaolianhe'+'fang'+'yao'+''AND
SU='Endometriosis'+'Chocolate cyst'+'EMs' AND FT='random'

Wan fang Data

Title or keywords:(zhenjiu or zhenci or zhenyaolianhe or tang or fang or zhenyao or zhongyao) and Title or keywords:(Endometriosis or Chocolate cyst or EMs) and ALL:(random)

Chinese Scientific Journal Database (VIP)

M=(zhenjiu OR zhenci OR dianzhen OR zhongyao OR zhenyaolianhe OR tang OR fang) AND M=(Endometriosis OR Chocolate cyst OR EMs) AND (U=random)

China Biology Medicine (CBM)

("zhenjiu"[Common field: Intelligence] OR "zhenci"[Common field: Intelligence] OR"dianzhen"[Common field: Intelligence] OR "zhongyao"[Common field: Intelligence] OR"tang"[Common field: Intelligence] OR "zhenyaolianhe"[Common field: Intelligence] OR "fang"[Common field: Intelligence] AND ("Endometriosis"[Common field: Intelligence] OR "Chocolate cyst"[Common field: Intelligence] OR "EMs"[Common field:Intelligence]) AND ("random"[All fields: Intelligent] OR "Randomized ControlledTrial"[Unweighted: Expansion])
